# #Springwatch #WildMorningswithChris: Engaging With Nature *via* Social Media and Wellbeing During the COVID-19 Lockdown

**DOI:** 10.3389/fpsyg.2021.701769

**Published:** 2021-10-13

**Authors:** Shi Xu, George Murrell, Sarah E. Golding, Beth F. T. Brockett, Birgitta Gatersleben, Caroline Scarles, Emma V. White, Cheryl Willis, Kayleigh J. Wyles

**Affiliations:** ^1^School of Hospitality and Tourism Management, University of Surrey, Guildford, United Kingdom; ^2^School of Psychology, University of Surrey, Guildford, United Kingdom; ^3^Natural England, Sheffield, United Kingdom; ^4^School of Psychology, University of Plymouth, Plymouth, United Kingdom

**Keywords:** COVID-19, natural world, wellbeing, online engagement, Facebook, thematic analysis

## Abstract

It is widely understood that nature engagement benefits human wellbeing. Such benefits have been found for real as well as virtual engagements. However, little is known about the role of nature-based videos in social media on wellbeing. With COVID-19 restrictions limiting people’s direct engagement with natural environments, this study critically examined people’s reactions to nature videos posted on Facebook during the first UK COVID-19 lockdown in 2020. Data consisted of comments on videos containing highlights from the British Broadcasting Corporation’s (BBC) Springwatch 2020 television series, and from a UK television presenter and naturalist’s (Chris Packham) livestream videos, posted on Facebook from March to July, 2020. Looking at the quantitative profile of a range of videos (i.e. views, likes and shares) and a detailed analysis of the 143,265 comments using thematic analysis, three major themes were generated as: (1) engaging with nature *via* social media is emotionally complicated, (2) cognitive and reflective reactions are generated from social media nature engagement and (3) engagement with nature-based social media as a mechanism for coping with stress during COVID-19. These findings inform understanding of how nature-related social media content and associated commentary have supported wellbeing throughout the ongoing pandemic and their importance as a means of continued support for wellbeing.

‘We feel that the injection of wildlife into people’s homes, particularly at this point, would be really valuable and uplifting’.

— Chris Packham, 2020

## Introduction

Human experiences of nature represent a multidisciplinary topic of increasing importance to researchers. Nature experiences are studied in a wide range of environments, from small urban gardens to dramatic forest landscapes (e.g. Natural England, 2020a,b) and occur at different levels of intensity, ranging from brief encounters with nature to longer term interactions (Bratman et al., 2015). These engagements have an array of positive outcomes on our physical health, such as reductions in heart rate, cortisol levels and blood pressure (Twohig-Bennett and Jones, 2018).

There is an increasing amount of evidence illustrating the various positive wellbeing outcomes (psychological health) that can be achieved from contact with the natural environment. For example, spending time in nature benefits our mood (McMahan and Estes, 2015). This includes increased positive affect (e.g. heightened levels of happiness; MacKerron and Mourato, 2013), self-transcendent emotions (e.g. awe, gratitude and wonder; Neill et al., 2018) and reduced negative affect (e.g. decreased feelings of anger and sadness; Bowler et al., 2010). Brief encounters with nature also have significant effects in reducing stress (Jiang et al., 2016; Hunter et al., 2019). These examples indicate hedonic wellbeing (e.g. Huta and Waterman, 2014; Disabato et al., 2016), focusing on happiness that stems from pleasurable, enjoyable experiences with limited discomfort (Huta and Waterman, 2014).

Other noteworthy psychological impacts include the ability of nature engagement to provide people with meaning and purpose in life (O’Brien et al., 2010), improve levels of life satisfaction (Ambrey and Fleming, 2013) and increase feelings of autonomy (Weinstein et al., 2009). These examples are related to eudaimonic wellbeing, defined as ‘the belief that wellbeing consists of fulfilling or realizing one’s daimon or true nature’ (Ryan and Deci, 2001, p.143), which is related to a higher and broader level of functioning and personal development (Huta and Waterman, 2014). There are also cognitive benefits associated with nature engagement (Bratman et al., 2012) including increased attention (Berto, 2005), memory span (Berman et al., 2012), positive and reflective thinking (Schertz et al., 2018), knowledge retention (Holden and Mercer, 2014) and decreased rumination (Bratman et al., 2015).

Of particular interest to this paper is the evidence of different types of virtual engagement with nature as providing wellbeing benefits. For example, improvement in mood has been observed after experiences in virtual reality settings which depict natural environments (Browning et al., 2020a; Yeo et al., 2020) and after viewing photographs of nature (Bowler et al., 2010). Reductions in stress levels have been associated with simulated natural environments (Kjellgren and Buhrkall, 2010), and Scarles et al. (2020) illustrated the wellbeing benefits of virtual reality simulations of natural environments in residential care environments. Alternatives to real-life nature therefore seem to aid wellbeing, although it is important to note that the effects of actual nature engagement on wellbeing seem to be stronger (McMahan and Estes, 2015; Browning et al., 2020b). Social media can be used to share and experience nature virtually (Wood et al., 2013; Hausmann et al., 2018) and has a large potential audience. For example, Facebook has over 2.7 billion users (Tankovska, 2021). However, research into this virtual engagement with nature is embryonic and gaps in knowledge exist, especially regarding the possible association between wellbeing and virtual nature engagement *via* social media channels.

In March 2020, in response to the COVID-19 pandemic, the UK government, like many other countries across the globe, implemented a national lockdown, imposing restrictions on the temporal and spatial limits of people’s movements (GOV.UK, 2020; The Health Foundation, 2021). During this lockdown, alternatives to real-life nature engagement were popular, such as watching and engaging with nature-based videos *via* social media. The People and Nature Survey for England (Natural England, 2020a) found that 41% of adults posted online content related to the environment during April to June 2020 (when social distancing measures were in place in the UK; including restrictions around green space use, limiting outdoor exercise during some of this time and shielding for vulnerable groups; The Health Foundation, 2021). Groups that were more likely to post environmental content were those with children, those who were aged 25–39, those with a university degree and those in employment.

Chris Packham, a UK wildlife presenter and naturalist, posted a series of nature-related livestream videos on his public Facebook page throughout the first lockdown in the UK (Packham, 2020). Packham also presented the BBC’s Springwatch (2020) on the television channel, BBC2, from which video clips with highlights of the programme were taken and posted on the BBC’s Springwatch (2020) Facebook page. Both Packham’s own videos, as well as the Springwatch videos, typically showed scenes of wildlife, landscapes, do-it-yourself videos of nature-based activities (e.g. making a garden pond) and nature-related discussions, filmed around Packham’s and other nature presenters’ homes (e.g. Gillian Burke in Cornwall and Iolo Williams in mid-Wales). Discussing the perceived importance of these videos, Packham said ‘This Springwatch will be a series like no other. As the country experiences lockdown, the natural world offers solace to so many’ (Packham, 2020).

With COVID-19 restrictions in place, as well as people engaging with nature-based content *via* social media, the UK national lockdown in Spring 2020 presented an opportunity to investigate the research gap regarding how people engage with nature *via* social media and whether there are associations between this form of simulated nature engagement and wellbeing. The current research aimed to examine this relationship by analysing the comments posted in response to videos on two Facebook pages: the BBC’s Springwatch, (2020) page and presenter Chris Packham’s own page. The comments from these two sets of videos were selected for two reasons. First, they were among the earliest in the UK to attempt to help the public engage with nature during lockdown. Second, they are frequently visited social media sites about nature.

## Materials and Methods

Data consisted of comments about the BBC’s Springwatch, (2020) TV series (68 pre-recorded videos posted on Facebook between 20 May and 18 June 2020, with 13,701 comments in total), referred to as ‘Springwatch videos’ and Chris Packham’s livestream videos (55 videos posted on Facebook between 18 March and 10 July, with a total of 129,564 comments), referred to as ‘livestream videos’. This time period was chosen to capture the first lockdown period in the UK, which started in March and was eased in July 2020. Analyses were conducted on all available videos from these two pages during these timeframes. We first conducted basic quantitative descriptive analyses on the number of views, comments, shares and reactions (e.g. likes and loves) per video. Next, we applied an inductive approach to identify patterns (or themes) based on the comments from the Facebook audiences. Qualitative thematic analysis was conducted using NVivo 12 to code data and actively generate themes. Thematic analysis is a ‘method for identifying, analysing, and reporting patterns (themes) within data’ (Braun and Clarke, 2006, p.79). This type of qualitative analysis has also been applied to public media communication of nature information (e.g. Wu et al., 2018) and is a widely used approach to analysing qualitative data. In this study, thematic analysis was used to analyse the public Facebook comments. To ensure rigour, coding was conducted independently by two authors. This included independently identifying the codes and assigning these into initial themes and sub-themes. Coding results were then compared, and discrepancies were reconciled *via* discussion (Brown et al., 1989; Tracy, 2010). The final set of codes and the relationships between them were then discussed within the wider research team, and three main themes were developed.

### Dataset 1 ‘Springwatch Videos’: Comments From the BBC’s Springwatch 2020 Videos

A total of 13,701 comments on 68 pre-recorded Springwatch videos were analysed. These videos ranged from montages of peaceful moments in nature captured in various UK locations and ‘mindfulness moments’, to guided tutorials of do-it-yourself projects to encourage wildlife into people’s gardens. The Springwatch Facebook page also posted a range of short wildlife documentaries ranging from 15s to 21min, which captured various UK habitats, including rock pools and chalk streams. The videos were all positive in tone, with some being more educational and others having a more humorous element to them. The clips were mainly pre-recorded and used voice overs or instrumental music. However, some posts also featured live camera recordings of wildlife with minimal audio added. All the posts allowed audiences to comment, and it is these comments which were included in our analysis. BBC Springwatch (2020) on Facebook provides an illustrative insight into the range of video content that was presented by BBC Springwatch.

### Dataset 2 ‘Livestream Videos’: Comments From Chris Packham’s Livestreams

This dataset consisted of 129,564 comments on 55 Chris Packham livestream videos posted on Facebook between March to July 2020. Chris Packham has a public Facebook page on which livestream (real-time) videos were frequently posted with his stepdaughter, Megan McCubbin, throughout lockdown and into the summer (see Packham (2020), for example screenshots). These livestream videos, titled #WildMorningswithChris, lasted approximately 45min each. The videos were educational and focused on different subjects, including general conversation about different types of wildlife, raising awareness on different environmental issues and demonstrating different nature-based activities, such as painting outdoors, wildlife photography and bird watching. The videos featured several different presenters who talked about their own areas of expertise, but also answered nature-related questions posted by the audience at the end of the videos. As such, audience engagement and interaction became a central aspect of these social media presentations.

## Results

### Descriptive Statistics for Both Datasets

Table 1 shows the number of posts, shares, likes and comments on the videos. The videos collectively received over 25 million views, although there was considerable variation in the viewing rates for the different videos (from 1,100 times to almost 3 million times). The videos from both datasets received a total of 143,265 comments, again with significant variations between videos. The highest number of comments in the Springwatch videos was for a video titled ‘the importance of wasps’ (posted on 07 June, 2020), featuring Chris Packham, which had 1,251 comments, while the fewest number of comments on a video was 14 on the ‘world oceans day – how to watch grey seals’ video (posted on 08 June, 2020). The highest number of comments in the livestream videos was for Episode 51 (posted on 15 May, 2020), which received 4,950 comments, while the fewest was for Episode 7 (posted on 20 March, 2020), with 308 comments. The average number of views and shares was lower for the livestream videos than for the Springwatch videos, but the number of active interactions (reactions and comments) was higher for Packham’s livestream videos.

**Table 1:** Descriptive statistics for the online engagement with the 68 and 55 videos examined in the Springwatch and livestream videos, respectively.

		Sum	Minimum	Maximum	Mean per video	Std. Deviation
Springwatch videos	Views	17,735,000	1,100	2,900,000	260,809	500,042
	Shares	54,717	81	14,000	805	1,773
	Reactions	219,350	610	33,400	3,225	4,384
	Comments	13,701	14	1,251	201	224
Livestream videos	Views	8,115,900	27,900	580,900	147,562	91,318
	Shares	23,402	58	6,674	452	897
	Reactions	221,756	956	19,000	4,032	2,618
	Comments	129,564	308	4,950	2,356	825

### Qualitative Analysis

**Figure 1:**
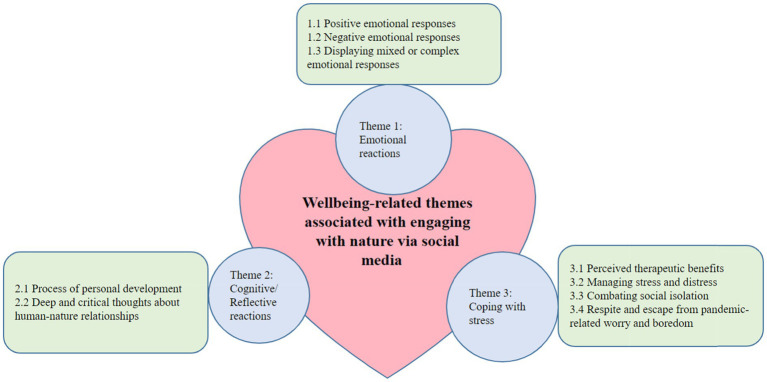
Thematic map of wellbeing-related themes associated with engaging with nature *via* social media during lockdown.

The qualitative findings of this study are derived from both datasets and combined. We generated three main themes from these comments: ‘Emotional reactions associated with nature engagement *via* social media’, ‘Cognitive and reflective reactions associated with nature engagement *via* social media’ and ‘Use of nature-based social media to cope with stress during COVID 19’. See Figure 1 for an overview of these themes, with their associated sub-themes, and see Table 2 for illustrative quotes from both datasets for each theme and sub-theme. The themes and sub-themes will now be discussed, along with additional illustrative quotes drawn from both datasets.

**Table 2:** Main themes and sub-themes with supportive example quotes.

Main themes	Sub-themes	Illustrative quotes (Dataset 1, Springwatch)	Illustrative quotes (Dataset 2, Chris Packham)
1. Emotional reactions	1.1 Positive emotional responses	My heart was singing for joy seeing those fox cubs playing on the trampoline. One of the most beautiful things I have ever seen in animal videos. 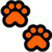 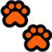 x	Thank you for bringing the birdsong and nature to us so uplifting
		The sound of birdsong and running water so calming	I love your banter Chris and Meg. Thanks for making me chuckle this morning 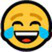
		Beautiful, love great crested grebes especially with babies (humbugs) so cute	I just love the pair of you, I sit here with a smile on my face for half an hour each morning! Thank you
	1.2 Negative emotional responses	It was my job to stand by the windows and kill every wasp that came in…I think I realised everything only has one life and it’s not mine to take. Shame I didn’t realise that about farm animals until 3years ago 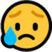	My daughter found a bumblebee yesterday, it was distressed and falling over + going round in circles. Obviously been poisoned 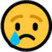 . I didnt know how to help it out of its misery
		Please don’t reveal his (badger) location. Someone will want to shoot him! 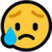 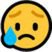	I’m getting very worried as we usually have lots of Beautiful bird’s in our garden, but we've noticed we've had none over the last couple of days 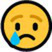
		Makes me sad for deaf people and that they can’t hear the beautiful birds singing or any other natural sound	I’m with you - my 89year old mum also has COPD [Chronic obstructive pulmonary disease] and her boiler broke down last week- I am worried
	1.3 Displaying mixed or complex emotional responses	You are so lucky to have a wonderful wood to go to. I’m 82 and been isolated for 9weeks, I love the woods but can’t go	The celandines remind me of walks along the cliff tops in Shanklin with my mum as a small child. Most of the cliff path fallen away. It was a magical place then. In the 50’s
		It takes me back to my childhood when every time I saw my Grandad at this time of the year he asked me if I had heard a cuckoo. I seem to recall in those days I heard one every year and now it is a treat to hear one	My mum, who passed in Dec, suffered dementia and mishear words, when told there were blue tits in the garden she thought they were blinking gits. I can never see a blue tit now without that memory, love her 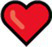
2. Cognitive and reflective reactions	2.1 Process of personal development	Oh wow, I never even noticed that before… now, when a kingfisher decides to show itself to me I will know the difference 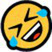 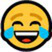	That was SUCH a fun and informative broadcast! Fascinating to learn the order of breeding, for birds. I witnessed 2 robins mating, very quickly on our fence, yesterday. Felt privileged, if a little voyeuristic for a second or two! 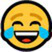
		I have a big fear of wasps but actually that was really fascinating to watch, I didn’t know that at all. I will appreciate them from a far from now. Really do enjoy learning about nature	I literally knew nothing about primroses even thought we have some in the garden - so thanks so much for this informative inspirational talk. Nature is super amazing - thanks for sharing your knowledge
	2.2 Deep and critical thoughts about human-nature relationships	…If lockdown has taught me anything… it’s been here for years and years and it’s now we fight to ensure it’s here for many generations to come	If we listened and watched nature a little more we wouldn’t have many of the struggles we as humans suffer I’m certain.
		We need to be there for nature after this. If we look after nature, nature will look after us	I am really feeling the climate crisis that is happening due to unnatural adversities. On the positive I have seen a good number of bees so far this year, and it was great to see the Red Admiral looking strong, so that feels positive and it is great to see wildlife holding on. I hope in the future…that more people start to make their gardens more wildlife friendly, because this would make a huge difference if we all managed that for the future of the natural world in which we live. It is positive that more people are taking more of an interest now, and I hope this continues and develops.
3. Coping with stress	3.1 Perceived therapeutic benefits	Watching Springwatch has been one of the most therapeutic things for me throughout this whole experience. Nature and all the beautiful creatures we share this planet with has brought me so much comfort during this surreal time that we are living through	The sound of the wind passing through the trees and leaves is beautiful and very therapeutic 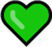
		The mindfulness moments have all been wonderful I always look forward to them, it shows that just watching and listening to nature, no added music, no commentary, is the very best tonic	Love these live streams, finding solace in nature 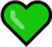
		Such a wonderful tonic, food for the soul. Thank you so much for these mindfulness moments and indeed for the whole of Springwatch. So very special 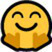 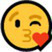	
	3.2 Managing stress and distress	Love the show helps me relax from the horrible anxiety this virus is given me	My anxiety has worsened over the last few weeks, but listening to you has lifted my moral
		I’ve watched Springwatch since it started and think this series has been wonderfully produced. As someone who suffers with social anxiety I’ve loved the focus on mental health issues and mindfulness of nature. I’ve been locked down due to health issues and have only been out to ho go shopping where I’d normally be out in nature. Luckily, our flat backs onto school fields surrounded by trees and hedgerows so I’m still getting to see and hear nature. We have seen, Jackdaws, Oystercatchers, Wrens, Robins, Thrushes, Black birds, butterflies, bees and think we have some reed warblers in the long grasses around the edge of the field. Thank you for bringing ginger nature to us when we can’t visit it by ourselves	Thank you so much guys, you are my go to in the morning and you’re really helping to curb my anxiety
	3.3 Combatting social isolation	Helps us who are isolating to still be connected to the great outdoors. A big thank you to everyone involved	I’ve watched every livestream. It’s an integral start to my day. Reading other people’s comments make you feel less isolated. Great show
			Good morning from Hull, so brilliant to feel so connected with everyone 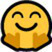 thank you Chris
	3.4 Respite and escape from pandemic-related worry and boredom	Well done to the Springwatch team! Brilliant series once again and under difficult conditions. This has been my break from the tedium of lockdown. Thanks for the memories and relaxation	Fantastic break to reality just what we needed, can’t wait for the next one
		I’ve had the best spring meandering through some of the ancient pathways across our part of the South Downs. Every walk we spot something new, every walk shows us something breathtaking. It helps distract from the terrible plight we are in for a precious hour or two, as do the marvellous Springwatch shows, thank you	Thanks Chris, a welcome break from all the doom and gloom! x
			You give us respite from the madness. Being able to join you in your garden albeit virtually is such a pleasure

*Exact quotes are used, including commenter typos. Emoticons are also presented exactly as commenters used them*.

### Theme 1: Emotional Reactions Associated With Nature Engagement *via* Social Media

The first theme presents a range of emotional reactions in the commenters’ responses to videos. The wide variations in these emotional responses to these videos are discussed across three sub-themes: ‘Positive emotional responses’, ‘Negative emotional responses’ and ‘Displaying both positive and negative emotional responses.

#### Sub-theme 1.1. Positive Emotional Responses

The first sub-theme relates to the positive emotional responses and hedonic pleasure that were expressed by commenters. Many positive feelings were coded in both datasets, including joy, astonishment, delight, having fun and feeling uplifted, moved and calmed. Commenters also used various emoticons (i.e. digital icons used to express ideas or emotions) to express their positive feelings. In particular, commenters often expressed feelings of joy and astonishment; for example, in response to a Springwatch video named ‘A totally amazing mother’, which featured a stoat, one commenter said as: ‘I’m really astonished by this little stoat! How quick and determined she is to feed her family! Poor rabbit and poor birds but it’s nature! A really nice video!’. Another person described the videos as being fun to watch, commenting on the ‘Cornish shags are daring thieves’ video from Springwatch: ‘That was such a great, montage of the Shags WOW!!… What beauties they are 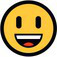, and what cheeky little thieves, they are stealing their neighbours twigs, for their own nest, 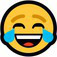 That was so funny!! 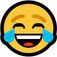 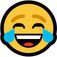
I, so loved watching them. 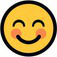

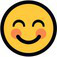
 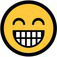

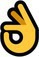

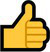
, 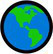 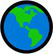 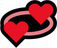 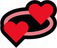, 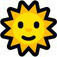 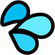
’. It can be inferred from the quotes that the emoticons reinforced the emotion/experience shared in the comments and can help increase social presence and leverage intimacy. In addition, the use of emoticons can enhance message usefulness as well as aid personal expression (Huang et al., 2020).

Additionally, there were a significant number of comments referring to the creatures’ cuteness, which appeared to generate pleasure, such as ‘awwwwwww it’s so cute!!! I want one!! 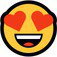

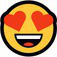

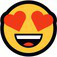

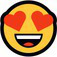

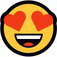
’ in reference to a hedgehog in the video ‘Meet the hedgehog lady’. These types of comments used the words ‘cute’ and ‘adorable’. The Facebook users appear to feel affection for these animals, which may be based on their appealing physical characteristics (Gunnthorsdottir, 2001).

In contrast to the Springwatch videos, which involve pre-recorded content, the livestream videos allowed for more interaction and engagement in the comments, as audience interaction was encouraged during the livestreams. Some of these comments also expressed positive emotions and commenters reported feeling amused, entertained, lucky and inspired. For example, some mentioned that the livestream was amusing and entertaining, which made them chuckle (see Table 2). Some commented that they felt lucky because they live close to nature and can walk with children and dogs easily; in contrast, others commented that the broadcasts were able to cheer them up despite them being unable to connect with nature physically. For example, one said as: ‘you and Megan cheer me up so much in this self-isolation xx’. Some commenters also wrote that the broadcasts had been inspirational as: ‘You inspired me to put out the bird feeder (it was put away when squirrels got into it). We have had some customers already’.

#### Sub-theme 1.2. Negative Emotional Responses

The comments posted in response to both sets of videos provide evidence that people also experienced negative emotions while watching the videos. Bhullar et al. (2013) and Knobloch et al. (2017) suggest that such negative emotional responses are often overlooked in our understanding of nature engagement. Spontaneous emotions of disgust or fear appeared to be evoked by a Springwatch video ‘Dance of the wolf spider’; one tagged a friend and said as: ‘eek!’ and the friend replied as: ‘omg I’m never going to sleep again’
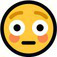

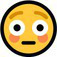
. Aversions to spiders or crawling insects may be due to disgust sensitivity as these invertebrates may produce a disgust response (Bixler and Floyd, 1999). Another example of a negative emotion is shown in response to the video ‘Importance of wasps’ in Springwatch: ‘I’m terrified of wasps, they always seem to pick on me and over the years despite trying to avoid them I get stung, usually when I don’t know they are there!’. This highlights that fear-evoking stimuli may be present in a natural environment, which can reduce motivation to engage with nature, as people tend to engage more in an environment when they perceive it is safe (Andrews and Gatersleben, 2010).

Reflecting the range of negative responses across both datasets, some commenters also revealed sad and sympathetic emotions: for example, the videos made some commenters sad for some groups, such as deaf people, because they cannot hear natural sounds (Table 2). Some audience members also expressed sad emotions relating to their inability to visit family for the foreseeable future. Thus, it suggests that nature imagery can trigger increased sensitivity and awareness of current personal circumstances. People also reported feeling guilty and expressed self-blame for their past behaviours in relation to nature. For example, ‘When I was in secondary school…I wasn’t very keen on maths so it was my job to stand by the windows and kill every wasp that came in… I realised everything only has one life and it’s not mine to take’.

#### Sub-theme 1.3. Displaying Mixed or Complex Emotional Responses

In addition to the clearly positive or negative emotional responses, some commenters also showed ambiguous or complicated emotional responses. In some instances, people’s comments included both positive and negative emotional responses to the same video. For example, while watching the ‘Importance of wasps’ Springwatch video, one person commented as: ‘This was really interesting. I always thought they [wasps] had no purpose. Still don’t like them though’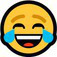. This commenter dislikes wasps but still displayed a positive feeling: interest. Another commenter expressed a mix of emotions in response to the livestream videos, including concern around not being able to self-isolate, while also expressing gratitude for the videos: ‘Morning Chris. Would love self-isolate but unfortunately I can’t. I do domestic help and look after elderly people. So I have to try keep myself protected the best I can. But thank you for these amazing educational videos, they cheer my day up! Look after yourself. X:)’.

Some commenters expressed envious emotions towards the presenters. For example, when Chris Packham was presenting around his cottage house and heard a cuckoo, someone commented as: ‘You are so lucky having a woodland on your doorstep’. Some commenters expressed nostalgia, a sentimentality for the past. For example, in the video of ‘dragons of the pond’, one relates aspects of the video to childhood memories: ‘…the smell of vine tomatoes always reminds me of my grandad [*sic*] shed when I was a kid’.

### Theme 2: Cognitive and Reflective Reactions Associated With Nature Engagement *via* Social Media

A second major theme generated through analysis of the comments relates to the audience’s cognitive and reflective reactions to the educational aspect of the videos. These reflections suggest some deep thinking. We identified two types of responses from the comments, which formed two sub-themes. The first sub-theme encompasses the ‘Process of personal development’, represented by reflections about what commenters felt they had learnt from watching the video. The second sub-theme focuses on ‘Deep and critical thoughts about human-nature relationships’, evidenced by reflections about subjects that were beyond the immediate content of these videos.

#### Sub-theme 2.1. Process of Personal Development

While engaging with nature-based content *via* social media can provide the opportunity to comment and share emotional responses, it can also lead to personal development through informal education and insight, which provides opportunities for learning and reflection. The educational aspect of learning new knowledge and skills could enhance people’s relationship with nature and their sense of connection to nature (Schwartz and Lederman, 2002). This is related to eudaimonic wellbeing, which is associated with mastery experiences. Mastery is defined as the engagement in activities that allows one to develop and grow, gain new skills or interests (Newman et al., 2014). Mastery experience during leisure time is associated with learning opportunities and provides internal psychological resources, including confidence, self-efficacy and expertise (Rieger et al., 2014). In one Springwatch video, ‘How to tell kingfishers apart’, commenters implied gaining the new skill of identifying the differences between female and male kingfishers. In one of the livestream videos, a guest presenter was invited to show how to draw birds with a pencil. In response, one commenter wrote, ‘You make it look so easy to draw!’, and some mentioned that they intended to purchase books on how to paint and draw birds.

Commenters also expressed that they were learning new knowledge from the videos, such as gaining information on dippers, a short-tailed bird with dipping movements. Some audience members for livestream videos mentioned the accessibility of the information presented as: for example, ‘Thank you for sharing information in an interesting and easily accessible way, Chris - you have increased my interest in natural history x’. In comparison with the Springwatch videos, the nature of Packham’s livestreams meant there was a significant focus on more informal education and knowledge sharing between the presenters and the audience. This included, for example knowledge of a tawny owl’s skull and how to feed birds.

#### Sub-theme 2.2. Deep and Critical Thoughts About Human-Nature Relationships

In addition to the educational elements of the videos, people also had wider reflections beyond the immediate content of the videos (e.g. thoughts about the past or future). Commenters discussed the meaning and value of nature to humans and how we should be more oriented towards the protection of nature in the future. Meaning can be understood here to be about finding a value or purpose in life, taking a long-term perspective (Newman et al., 2014). The comments included statements about nature being an essential part of human life and how humans must live alongside nature in a sustainable way and develop a symbiotic relationship. Some commenters in Packham’s livestreams showed positivity and optimism towards the state of nature in the future: ‘we … think that something good and positive to nature will come out of all this. Rethink what is important’ and ‘…In a world of tough times - spread the love and positivity - for she (the earth) is listening’
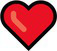

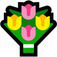

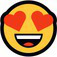

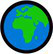

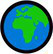

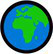

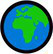

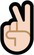
. Some audience members mentioned that the livestreams gave them ‘time to reflect on the beauty of our planet’ and that they were ‘hopeful for a bright future’.

However, as well as positive reflections, some audience members also expressed pessimistic thoughts about humankind’s relationship with nature: ‘After watching springwatch it occurred to me how dreadful it would be if the Covid-19 virus had affected all the wildlife rather than us humans?’. Some expressed concern about the wildlife presented and hoped that things would get better. One wrote as: ‘If we listened and watched nature a little more we wouldn’t have many of the struggles we as humans suffer I’m certain’. Commenters also discussed issues of climate change and what humans could do to stop wildlife vandalism and reduce our environmental impact on the planet: for example, ‘Please help and do what you can to save our precious trees and to stop the destruction, so crucial now more than ever in these times of runaway climate change…’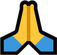.

The videos also opened up reflections beyond nature and COVID-19, with some of the comments indicating wider socio-environmental debates. For example, some commenters mentioned their concerns that HS2 (HighSpeed2; a major railway infrastructure project in the UK) could damage beautiful countryside and wildlife. They suggested that policy makers should redirect resources currently being invested in HS2 towards positive environmental efforts. One commented as: ‘whilst we are all enjoying hearing the dawn chorus, HS2 contractors are currently destroying ancient woodland and thousands of bird nests and other wildlife will be destroyed during nesting season…It’s like us coming home and finding our homes burnt to the ground with our families still inside… 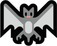

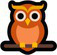

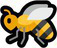

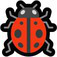

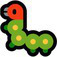

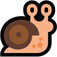

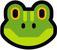

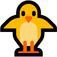
. ITS THEIR WORLD TOO’. This sub-theme shows that while some people were positive and hopeful about the future of human-nature relationships, others were frustrated about the current situation and felt that policy makers needed to be held to higher levels of accountability on the management of nature and the environment.

Not only did engagement with nature *via* social media trigger reflections on wider socio-environmental issues, such as climate change and biodiversity loss, it also provoked discussions on other societal issues, such as racism in relation to nature engagement. For example, in one livestream video, Packham discussed the incident of a white woman who called the police on a black birdwatcher and pretended to be threatened when she was dog walking in Central Park, New York. Commenters expressed distress that some dog owners apparently refuse to use leads: ‘Great you are doing a piece on dogs off leads. Not only are they are risk to the wildlife off the lead, they are at risk of accidents around farm land’. Other commenters expressed disgust at what was perceived to be racist behaviour by the white woman: ‘absolutely disgusting her behaviour’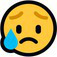. Some of the audience also discussed the importance of the ‘Black Lives Matter’ movement and expressed appreciation that the presenters were discussing racism and diversity issues when talking about nature experiences: ‘Thank you for raising BLM this morning. The more we educate the better society will be. How great you’ve used your profile for this’.

### Theme 3: Use of Nature-Based Social Media to Cope With Stress During COVID-19

In addition to the esssmotional and cognitive/reflective reactions, the commenters also reported that this virtual engagement with nature had helped them cope with the stressors derived from COVID-19, such as fear, anxiety and isolation. Research has shown that the fear and anxiety around a new disease and its potential consequences can be overwhelming, and coping with stressors in a healthy way can help people feel stronger (Dijkstra and Homan, 2016). We identified four aspects of using social media to cope with stress, presented as sub-themes: ‘Perceived therapeutic benefits’, ‘Managing stress and distress’, ‘Combatting social isolation’ and ‘Respite and escape from pandemic-related worry’. The overall theme is different from Themes 1 and 2, since it is directly related to the impact of COVID-19 on individuals.

#### Sub-theme 3.1. Perceived Therapeutic Benefits

The first sub-theme focusses on the perceived therapeutic benefits of the videos that commenters believe have improved their wellbeing during the pandemic. People discussed nature as being a salvation, a tonic, lifting the spirits and soothing their souls. The evidence for this was especially strong in the Springwatch videos. One person described Springwatch as a ‘beautiful jewel’ that brings the beauty of nature to the audience. Some commenters suggested the videos provided a therapeutic landscape which helped their wellbeing during COVID-19 (e.g. ‘A spring like no other in Springwatch): ‘It has been so therapeutic and calming to the mind in the troubles we have all had to endure. The power of nature is so healing!’. Some mentioned that the sounds and scenes of nature and moments of tranquillity in the videos contributed to this sense of a therapeutic landscape. Some even commented that the Springwatch videos should be available for NHS (National Health Service in the UK) patients, to help people with mental health issues. People also commented that listening to birdsong and walking with dogs was especially therapeutic to people’s wellbeing: ‘Birdsong is a pure tonic for the soul 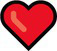
’ and ‘Dog walking is my saviour right now!’.

#### Sub-Theme 3.2. Managing Stress and Distress

The second sub-theme is focused on how engaging with nature *via* social media appears to be beneficial for commenters beyond being a general salve for wellbeing. Some commenters specifically wrote in terms of how the videos reduced their negative affect and helped them cope with mental health symptoms, such as anxiety and distress during the tough times of the pandemic. For example, due to COVID-19, a lot of university and school teaching was moved online during the lockdown in the UK, and some teacher commenters mentioned that these videos were much needed after a stressful day of teaching, helping them reduce feelings of anxiety. Some commenters who were facing physical or mental health challenges discussed how Packham’s livestream videos were important in helping to reduce their stress levels and lift their spirits. For example: ‘with ASD [autism spectrum disorder] here and my anxiety has been in overdrive despite my rational brain trying to look at this all logically. Thank you for bringing sanity back into the start of my day, going to try and get out into the garden and these blue skies and start getting some spring jobs done’. Commenters also discussed how the broadcasts can potentially have impact beyond the immediate viewer, for example: ‘Hi Chris and Megan…my daughter has mental health (issues) and this will help me to help her thank you’.

#### Sub-theme 3.3. Combatting Social Isolation

This sub-theme was especially evident in the comments on Packham’s livestream videos. Commenters on the livestreams demonstrated social interaction with other commenters, which they felt helped to combat social isolation during the COVID-19 lockdown. For instance, commenters greeted others and the presenters with comments, such as: ‘Morning all from Bournemouth! Hope you are all keeping safe and well’ and ‘Good morning everyone. It’s a beautiful day here in South Wales. Hope you all have a great day’. Many commenters discussed how watching the broadcasts had become part of their routine, how it had brought people together, created a community, enabled people to feel connected with others and helped them feel less isolated as a result. They also tagged their friends and shared good wishes. For example, one tagged a friend and said as: ‘seeing Chris and Meg sets the right mood for the day doesn’t it. Hope your days are as sunny as ours, makes things a little better to get through’. As a result, the comments indicate that many felt less lonely and isolated by engaging with these nature videos *via* social media and that they feel more connected to one another *via* a shared enjoyment of the posts. These findings suggest that livestreaming makes ‘this isolation bearable’ and brings people virtually together to appreciate nature when it is not physically possible to be together. This is associated with another eudaimonic wellbeing concept related to affiliation, which involves friendship, love and social connectedness (Ryff and Singer, 2008); watching and engaging with nature *via* social media can help combat loneliness and build a strong bond *via* an online community.

#### Sub-theme 3.4. Respite and Escape From Pandemic-Related Worry and Boredom

Montero-Odasso et al. (2020) suggest that constant exposure to issues related to the pandemic through media and everyday responsibilities can be overwhelming for many. People need respite and escapism from worry and anxiety brought about by changed circumstances, such as working from home, home schooling and caring for children, and for adults who are unable to access care support. Nature engagement *via* social media can therefore provide a virtual opportunity to escape by virtue of aesthetically rich experiences. Indeed, some of the audience mentioned that these videos had provided real ‘escapism’, by distracting them from worrying about COVID-19, which they reported as much needed. For example, one mentioned as: ‘It helps distract from the terrible plight we are in for a precious hour’.

Other challenges during lockdown may have included boredom for people who were not working, were on furlough, were shielding or were unable to engage in other activities, such as volunteering in the community. These videos helped them escape from those feelings of boredom. One person, for example mentioned that the videos were ‘such a light relief in otherwise mundane days’. Linked to sub-theme 2.1 (process of personal development), some commenters mentioned that the broadcasts had enabled them to grow their pre-existing wildlife knowledge and their book collection during lockdown, but that beyond this, the videos had also provided ‘an escape from the madness’.

## Discussion

Engagement with nature has been shown to benefit people’s wellbeing in many ways (e.g. McMahan and Estes, 2015; Twohig-Bennett and Jones, 2018). However, little research has investigated if and how virtual nature engagement *via* social media is associated with such wellbeing benefits. We analysed a total of 143,265 comments publicly available on Facebook, to address this gap in the literature. Our research showed that, at least among this group of commenters, watching and engaging with nature virtually *via* social media during COVID-19 lockdown can elicit a variety of hedonic wellbeing outcomes, such as positive emotions (e.g. feeling calm, relaxed, joyful, moved, uplifted and inspired) and feeling free to express negative emotions. It may help people feel more relaxed and support recovery from stress and mental fatigue. Virtual engagement with nature *via* social media may also help improve an audience’s eudaimonic wellbeing and give people a sense of meaning and sense of connectedness to nature and other people. It is seen as informative and meaningful to the human-nature relationship and enables people to interact with others and discuss their experiences, which helps build a strong bond in this online community. We also found that people reported virtual nature engagement *via* social media to be therapeutic, soothing, healing and useful for combatting loneliness. Thus, engagement with nature-based social media may support wellbeing in multiple ways and could aid coping during COVID-19. This study has provided new insights into the ways in which the benefits of nature engagement can be supported and achieved.

Overall, these findings and themes support the previous literature on nature engagement and wellbeing (McMahan and Estes, 2015; Twohig-Bennett and Jones, 2018). For example, interacting with natural environments can realise benefits, such as improvements in anxiety disorders and depression (Berman et al., 2012), something also suggested by our theme on coping with stress during COVID-19. Specific to the context of COVID-19, Labib et al.’s (2021) pre-print synthesis of the literature suggests that nature engagement during COVID-19 is associated with improved mental health, wellbeing, physical activity and sleep quality. However, they also point out that nature engagement could be related to greater COVID-19 infection and mortality rates if proper social distancing measures are not in place. Therefore, virtual alternatives, such as social media platforms, could help people engage with nature where being outdoors may not be as safe, for example during periods of high community transmission of COVID-19.

This study has deepened the understanding of the link between engaging with nature virtually *via* social media and wellbeing. Our results suggest that these experiences may be related to wellbeing on a broad level. A mixture of both hedonic and eudaimonic dimensions of wellbeing, as well as effective stress management, were experienced by the commenters and conveyed to their readership through social media. The themes identified in this study suggest that studies of wellbeing can be extended in a virtual nature engagement setting. This supports the calls by Cleary et al. (2017) for research that considers various forms of wellbeing and more studies exploring the nature connectedness−wellbeing relationship.

Consistent with Knobloch et al. (2017), results of this study show the complexities in the context of the audience’s experiences when they watch online content, and we need to caution against the oversimplification of their desire for joy and pleasure during nature engagement. Particularly, the link to deep and critical reflections and stress management shows the importance of how social media engagement experiences can go beyond the here and now. It is important to recognise that the audience’s experience is not only about enjoyment in the moment; their interactions *via* social media and associated experiences may contribute to further personal cognitive reflections and help them cope with stressors, as well as having the potential to impact the audience’s lives beyond the watching activities *via* social media. The informal education and knowledge sharing in the videos could also facilitate deep connections to nature and wildlife (Jarrahi and Sawyer, 2013).

Engagement with nature benefits wellbeing, but we know relatively little about people’s engagement with nature when access to outdoor spaces is restricted. This paper extends the current literature on nature and wellbeing in the context of COVID-19 by exploring the potential for virtual engagement with nature *via* social media. In general, the evidence from previous studies implies that engaging with nature has a strong positive health effect and positive influences on psychological wellbeing (White et al., 2019). Landscapes devoid of nature have less positive and in some cases negative impacts on wellbeing and health (e.g. Grinde and Patil, 2009; Hunt et al., 2016). The current research suggests that people may seek out other forms of nature engagement if their access to real natural spaces is restricted, including virtual engagement. They may like, share or comment on nature-related videos, which is a deep form of engagement, allowing the audience to be motivated to take advantage of a public forum that empowers them to voice their opinion (Dolan et al., 2019). It supports previous research (Browning et al., 2020a; Yeo et al., 2020) that engaging with nature virtually can have restorative benefits and build a sense of connectedness to nature. Natural environments are purported to contain fewer demands on cognition, compared to other environments, such as an urban environment, where the senses can be overloaded; exposure to nature can therefore enable restoration and recovery from stress (Ulrich et al., 1991; Kaplan, 1995). Such restoration has dual benefits, as it can both reduce negative affect and enhance positive affect (Berto, 2005; Van Aart et al., 2018). von Lindern (2017) suggested that a natural environment does not have to be physically distant to provide a sense of being away, but it must be meaningfully different. Naturalists, such as Chris Packham, have launched social media engagement initiatives for the audience to take the time to appreciate wildlife, which arguably makes it meaningfully different from merely staying at home. There is the additional benefit of creating a community *via* social media, which further supports wellbeing by reducing loneliness and increasing a sense of connectedness.

Better understanding the relationship between watching and engaging with nature virtually *via* social media and wellbeing-related outcomes has significant implications for promoting, designing and delivering (real and virtual) nature-based interventions. The commenters highlighted perceived benefits including positive emotions, reductions in social isolation and increases in social and cognitive functioning. Because of the restrictions on free movement during the COVID-19 crisis, the public’s engagement with natural environments has changed significantly (Natural England, 2020a). In the analysis, we found that for some people who live close to nature, watching nature *via* social media is an additional form of engagement. For others, however, nature engagement *via* virtual alternatives is their only method of nature engagement, because they could not visit nature physically and they lack exposure to restorative environments. It is important that people therefore recognise the potential benefits of engaging with nature through virtual alternatives, such as social media platforms. Public health interventions in future pandemics could involve virtual nature engagement *via* social media, given the potential wellbeing benefits indicated in this study. Future research should explore whether engaging virtually with nature *via* social media is indeed effective at maintaining or improving wellbeing outcomes among the general population during lockdowns and other forms of restricted movement. It is also possible that people considered clinically vulnerable during the pandemic, who were having to take extra precautions to stay at home and shield, may have benefitted from watching and engaging with nature virtually on social media platforms; this is another avenue for future research to explore.

This paper has its limitations. First, although we conducted thematic analyses of comments on Springwatch, 2020 and Chris Packham’s livestream video Facebook page posts, which were popular channels on nature and wildlife viewed by the public, other channels or social media platforms were not investigated. Non-nature videos posted by other high-profile figures were not included within this study, so conclusions about these findings in relation to non-nature videos cannot be drawn. It is not possible to definitively claim that these themes are specific to the nature-focused content of these videos. Future research could therefore study other channels and platforms, and the feeds of other public figures, to broaden the findings and explore potential differences in the reactions between videos with or without a focus on nature. Second, as this study is qualitative in its focus on social media comments on nature engagement, future studies could complement these findings by seeking to quantify which aspects of the videos may make them more or less ‘successful’ in generating a certain response in the audience. Researchers could also design quantitative studies which, for example survey commenter’s experiences of nature engagement *via* social media. Third, our study is limited due to the inability to identify demographic information about the commenters due to the nature of Facebook. While we do have a large sample size and a large number of comments, not much is known about commenters’ age, ethnicity, education, location, sexual orientation, etc. Therefore, it is difficult to claim generalisability of our findings. Having said this, it is not necessarily the goal of the qualitative research to be generalisable (Polit and Beck, 2010), as qualitative studies are meant to provide insights into a specific phenomenon, which for this study was watching and virtually engaging with nature *via* social media. Fourth, while emoticons were not analysed independently, the presence of emoticons reinforced the emotion/experiences shared in the comments. It would be pertinent for future studies to examine the role of emoticons and non-verbal communications *via* social media in communicating wellbeing. Fifth, while we focus only on nature engagement during COVID-19, future researchers are encouraged to compare pre-, during and post-COVID-19 nature engagement *via* social media. Finally, it should be acknowledged that there may be some discrepancy between how people actually experienced watching the videos and how they chose to represent these experiences in their public comments (Schlosser, 2020).

## Conclusion

Although there is a range of research exploring the positive wellbeing outcomes from humans engaging directly with nature (e.g. Berman et al., 2012; White et al., 2017), relatively little research has been conducted exploring wellbeing associated with virtual nature engagement *via* digital alternatives, such as watching nature videos on social media. To the best of our knowledge, this qualitative paper is among the first to explore the comments posted by members of the public who were watching and engaging with nature virtually on a social media platform. Drawing upon qualitative data, our findings suggest that such virtual nature engagement during the COVID-19 lockdown was perceived by social media commenters to be associated with various wellbeing benefits, including hedonic wellbeing (e.g. increased positive emotional reactions and feeling free to express negative emotions), eudaimonic wellbeing (e.g. process of personal development and affiliation to others online) and coping with stress during the pandemic.

## Data Availability Statement

The original contributions presented in the study are included in the article, and further inquiries can be directed to the corresponding author.

## Author Contributions

SX and GM contributed to conception and design of the study and performed the analysis. SX, GM, and SG wrote the first draft of the manuscript. All authors contributed to manuscript revision, read and approved the submitted version.

## Funding

This research was funded by the ESRC (Economic and Social Research Council) UKRI (UK Research and Innovation) COVID-19 fund to help support the UK Government Green Recovery Strategy from the global COVID-19 pandemic (code: ES/V005464/1).

## Conflict of Interest

The authors declare that the research was conducted in the absence of any commercial or financial relationships that could be construed as a potential conflict of interest.

## Publisher’s Note

All claims expressed in this article are solely those of the authors and do not necessarily represent those of their affiliated organizations, or those of the publisher, the editors and the reviewers. Any product that may be evaluated in this article, or claim that may be made by its manufacturer, is not guaranteed or endorsed by the publisher.
